# Hierarchical CoNiO_2_ Microflowers Assembled by Mesoporous Nanosheets as Efficient Electrocatalysts for Hydrogen Evolution Reaction

**DOI:** 10.3390/ma16062204

**Published:** 2023-03-09

**Authors:** Dingfu Zhang, Jiaxin Yao, Jinling Yin, Guiling Wang, Kai Zhu, Jun Yan, Dianxue Cao, Min Zhu

**Affiliations:** 1 Key Laboratory of Superlight Materials and Surface Technology of Ministry of Education, College of Materials Science and Chemical Engineering, Harbin Engineering University, Harbin 150001, China; 2 Technology Innovation Center of Industrial Hemp for State Market Regulation, College of Chemistry and Chemical Engineering, Qiqihar University, Qiqihar 161006, China

**Keywords:** hydrogen evolution reaction, synergistic effect, CoNiO_2_ nanosheets, electrocatalysis

## Abstract

In order to alleviate the energy crisis and propel a low-carbon economy, hydrogen (H_2_) plays an important role as a renewable cleaning resource. To break the hydrogen evolution reaction (HER) bottleneck, we need high-efficiency electrocatalysts. Based on the synergistic effect between bimetallic oxides, hierarchical mesoporous CoNiO_2_ nanosheets can be fabricated. Combining physical representations with electrochemical measurements, the resultant CoNiO_2_ catalysts present the hierarchical microflowers morphology assembled by mesoporous nanosheets. The ultrathin two-dimensional nanosheets and porous surface characteristics provide the vast channels for electrolyte injection, thus endowing CoNiO_2_ the outstanding HER performance. The excellent performance with a fewer onset potential of 94 mV, a smaller overpotential at 10 mA cm^−2^, a lower Tafel slope of 109 mV dec^−1^ and better stability after 1000 cycles makes CoNiO_2_ better than that of metallic Co and metallic Ni.

## 1. Introduction

Fossil fuels are running out and the planet is getting worse, meaning it is really important to develop high-power and energy density alternative energy devices. There is no doubt that hydrogen (H_2_) is a renewable and clean energy that can be obtained from the environment [1,2,3,4]. It is well known that hydrogen is the most abundant element on earth; currently, 90% of H_2_ is acquired from fossil fuels, leading to the aggravation of the energy crisis [5]. Electrocatalytic water (H_2_O) splitting, using H_2_O as the hydrogen source and powered by renewable electricity, is regarded as a potential H_2_ synthesis strategy to minimize carbon emissions [6,7,8,9,10]. The hydrogen evolution reaction (HER) is, however, still limited by the large overpotential that is found in splitting H_2_O, which makes the reaction performance hover at a significantly lower level. It has been shown that certain noble metals with the low overpotential, such as platinum-based materials, are expected to serve as excellent catalysts for hydrogen production in practical application, though the scarcity of these materials and the high price make them difficult to use in practice [11,12,13,14]. To solve HER problems, we need efficient non-precious metal electrocatalysts [15,16,17,18,19,20].

To date, non-precious metals as the substitute materials have been extensively explored for HER to replace precious metals. By virtue of their structure design and synergistic effect, catalysts are capable of introducing hydrogen into a system at a higher performance level. There is a possibility of increasing the electrochemical HER performance by increasing the number of active sites in nanosheet structures. For example, Ni_0.85_Se nanosheet on graphene owns many active sites and well HER catalytic property [21]. It may also be advantageous to improve performance by taking advantage of the synergistic effects of bimetallic electrocatalysts. Singh et al. reported that the B-TiZr-2.5 catalyst has improved HER activity [22]. It is recognized that the single metal catalyst is not enough to make the HER catalyst active. Therefore, other metal oxide phases need to be added to obtain higher HER performance.

It is well known that most transition monometal oxides have a very good catalytic activity, but due to their low active site and specific surface area, their further applications are limited. Due to this, many efforts have been made in order to further enhance the catalytic properties of transition monometal oxides. Among them, the introduction of bimetallic oxides is a strategy. Wang et al. reported that NiCo_2_O_4_ @ Ni(OH)_2_/NiOOH catalyst has the improved HER activity [23]. In view of the synergistic effect, developing the efficient Co-Ni oxide catalysts by a simple and cost-effective method is attractive and necessary for HER [24]. Herein, hierarchical CoNiO_2_ microflowers assembled by mesoporous nanosheets are synthesized successfully by a simple hydrothermal method together with an annealing process. Benefitting from the large specific surface area and synergistic effect between Ni-Co metals, this resultant CoNiO_2_ shows stunning catalytic properties for HER, such as the low Tafel slope of 109 mV dec^−1^, small onset potential of 94 mV, low charge transfer resistance and a long-term stable behavior regarding catalysis.

## 2. Experimental

### 2.1. Synthesis of CoNiO_2_

The hierarchical CoNiO_2_ microflower was prepared [25,26]; concretely, an ethanol-water (2:1 *v*/*v*) mixture containing 0.73 g Ni(NO_3_)_2_·6H_2_O, 1.45 g Co(NO_3_)_2_·6H_2_O and 1.4 g hexamethylenetetramine (HMT). This was then transferred into a 50 mL autoclave lined with Teflon after it had been stirred for 30 min. Upon sealing the autoclave, it was placed into an electric oven which had been set at 95 °C for 8 h. We first cooled down the as-obtained Co-Ni precursors under a vacuum to room temperature, washed with water and ethanol several times to remove the unreacted residues, followed by drying at 60 °C for 8 h under vacuum to ensure the precursors were completely dry. Lastly, CoNiO_2_ was synthesized following annealing at 450 degrees Celsius in an Ar atmosphere for two hours at 1 °C per minute. For comparison, the individual Ni and Co catalysts were procured by the same method without adding Cobaltous nitrate hexahydrate and Nickel nitrate hexahydrate, respectively.

### 2.2. Characterizations

In terms of surface analysis, X-ray Photoelectron Spectroscopy (XPS, ESCALAB250) is a practical method that can be used for both qualitative and quantitative analyses of solid surfaces, as well as structural identification. X-ray diffraction (XRD, Rigaku TTR III) is a research method to obtain information such as the composition of materials, the structure or morphology of molecules in materials. Scanning electron microscope (FESEM, FEI Quanta 200 FEG) can not only observe the surface morphology, but also analyze the components and elements. Transmission Electron Microscopy (TEM) and High-Resolution TEM (HRTEM) are instruments for imaging and analyzing the microstructure of materials (FEI Tecnai G2 F20).

### 2.3. Electrochemical Measurements

It took 550 μL of 6/5 (*v*/*v*) water/ethanol solution, 5 mg of CoNiO_2_ powder and 20 μL of Nafion (5 wt %) to make the mixed solution and this was sonicated mildly for 30 min. Then, 5 μL of the prepared catalyst solution was evenly coated on carbon fiber cloth with an area of 1 cm^2^. Typical electrochemical measurements were carried out using a three-electrode setup involving the working electrode (the loaded carbon cloth), as well as the reference electrode (the Hg/HgO solution) and the counter electrode (the carbon rod). According to the equation below, the potentials reported in this study have been converted to the reversible hydrogen electrode (RHE) scale and are now expressed as follows: E(RHE) = E(Hg/HgO) + 0.098 + 0.0592 × pH. The electrocatalytic experiments were carried out on an Autolab PGSTAT 302 (Eco Chemie) workstation.

## 3. Results and Discussion

**Figure 1:**
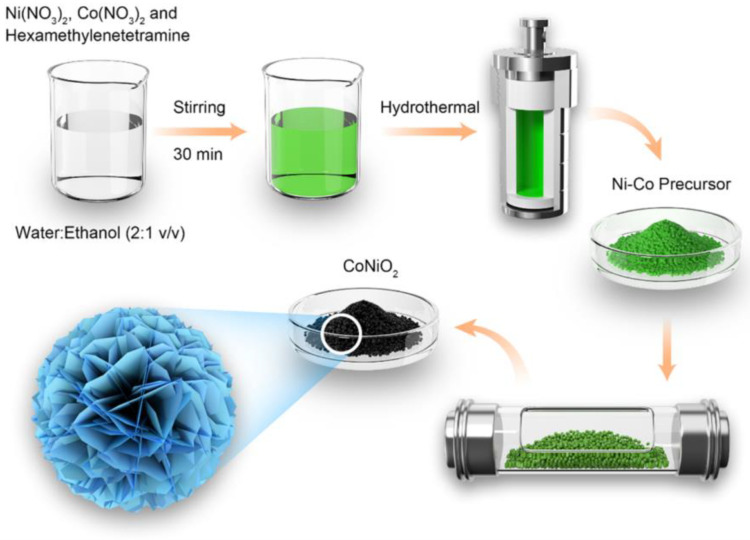
The fabrication procedure is illustrated in this schematic diagram for hierarchical CoNiO_2_ microflowers assembled by mesoporous nanosheets.

The synthetic method can be demonstrated visually in Figure 1, in which a two-step method had been used to prepare the typical mesoporous CoNiO_2_. Hydrothermal processes are characterized by the hydrolysis of ethanol and hexamethylenetetramine, forming carbonate ion and hydroxide ion, which are then reacted with Ni^2+^ and Co^2+^ to form precursors of nickel and cobalt hydroxy-carbonates during the reaction. Secondly, under the Ar atmosphere, the as-prepared Ni-Co precursor was transformed into mesoporous CoNiO_2_ with a hierarchical structure. Furthermore, individual Co and Ni catalysts were prepared so that the synergistic effect of these components could be evaluated.

**Figure 2:**
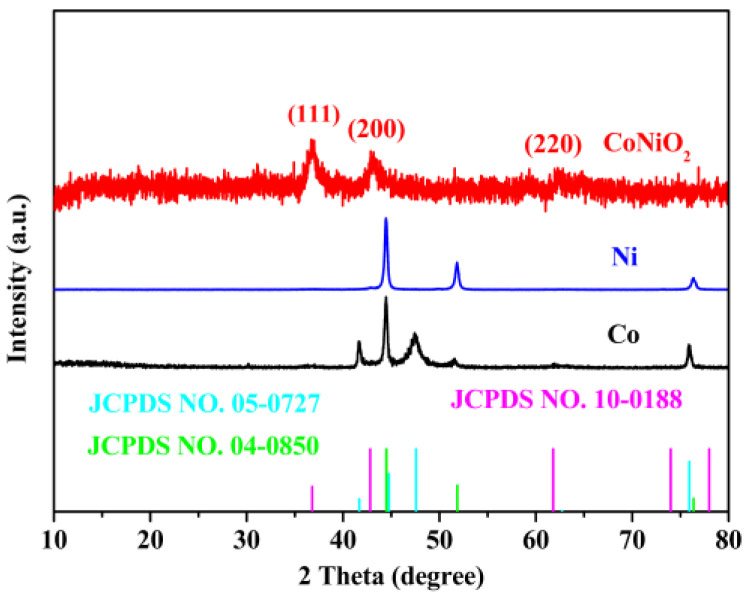
An XRD pattern showing the crystalline structure of CoNiO_2_, Ni and Co.

To investigate the crystal structure of the electrocatalyst, an XRD analysis has been carried out. The characteristic peaks of CoNiO_2_ in Figure 2 located at 36.8°, 42.8° and 61.8° correspond to the (111), (200) and (220) planes of the cubic CoNiO_2_ (JCPDS No. 10-0188) [25,26]. Meanwhile, the (111), (200) and (220) planes of metallic Ni can be matched to the diffraction peaks of Ni specimens at 44.5°, 51.8° and 76.4° (JCPDS No.04-0850) [27]. The characteristic peaks of the Co sample located at 41.7°, 44.7°, 47.6° and 75.9° are associated to the (100), (002), (101) and (110) of the metallic Cobalt’s planes (JCPDS No. 05-0727) [28]. It was observed that there were no significant peaks of impurities, which indicated that the products were phase pure. Particularly, compared with the single metallic Ni or Co specimen, CoNiO_2_ presents the weaker peak intensities. The relatively poor crystallinity means that CoNiO_2_ exhibits the thinner thickness and higher defects concentration, which is expected to show better activity in the hydrogen evolution reaction.

**Figure 3:**
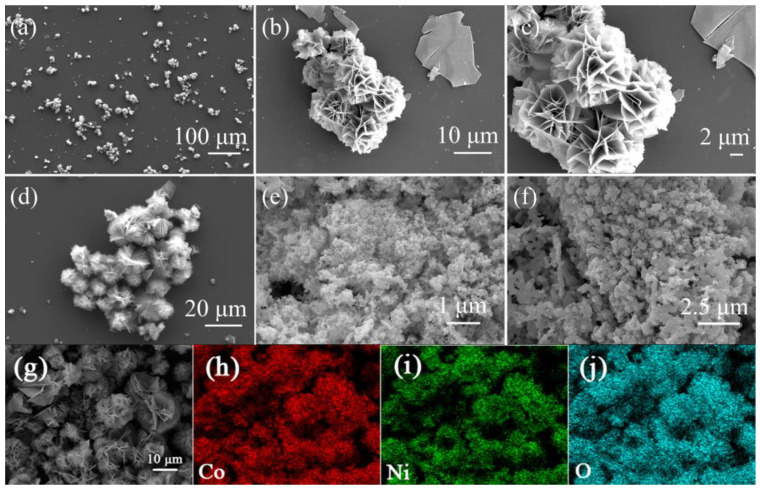
SEM images of (**a**–**d**) the hierarchical CoNiO_2_, (**e**) the metallic Ni and (**f**) the metallic Co. SEM picture of (**g**) CoNiO_2_ and (**h**–**j**) the homologous elemental mapping pictures of Co, Ni and O for CoNiO_2_ materials.

Electrocatalysts were characterized morphologically using scanning electron microscopy (SEM). As shown in the low-magnification SEM image (Figure 3a,b), the as-prepared CoNiO_2_ material presents the flower-like microsphere structure with an average diameter of about 9 μm. According to the high-resolution SEM images, it is also evident that the microflowers are a result of the two-dimensional thin nanosheets that are assembled into the microflowers (Figure 3c,d). For comparison, the morphologies of the metallic Ni and metallic Co were also provided, as shown in Figure 3e,f. It is worth noting that the individual Ni and Co samples are formed by spherical particles along with the severe agglomeration simultaneously. Having a regular and open hierarchical structure gives CoNiO_2_ a more specific surface area, makes electrolyte injection efficient and helps it be more catalytic. The conductivity of CoNiO_2_ material obtained after calcination is increased, which makes hydrogen evolution easier. The above results show that the prepared CoNiO_2_ material has a large specific surface area, exposed more active sites and good conductivity. Figure 3g–j shows the SEM picture and the elemental mapping pictures for CoNiO_2_ materials, indicating the good distribution of Ni, Co and O elements in the hierarchical flower-like nanosheets.

**Figure 4:**
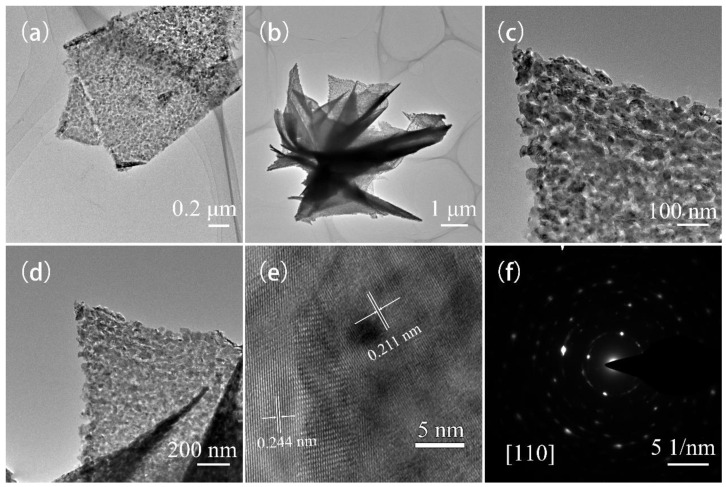
(**a**–**d**) TEM figures, (**e**) HRTEM figure and (**f**) SAED pattern of CoNiO_2_.

We investigated the morphology of the as-prepared CoNiO_2_ sample by transmission electron microscopy and high-resolution transmission electron microscopy in a further study. According to the TEM images of CoNiO_2_ shown in Figure 4a–d, the thin nanosheets are used to construct the microflowers, and therefore, TEM images are in agreement with SEM observations, where the nanosheets are assembled by thin walls. A distinctive characteristic of CoNiO_2_ microflowers is that they are formed by thin nanosheets of CoNiO_2_ over a thickness of approximately 100–300 nanometers. In Figure 4e, it is depicted that the lattice fringes with separations of 0.211 nanometers and 0.244 nanometers correspond to the crystallographic planes (200) and (111) for cubic CoNiO_2_, respectively. It is also worth mentioning that the SADE image exhibits a pattern along the zone axis of the [110] zone of cubic CoNiO_2_, which further confirms that CoNiO_2_ phase has been successfully generated (Figure 4f).

**Figure 5:**
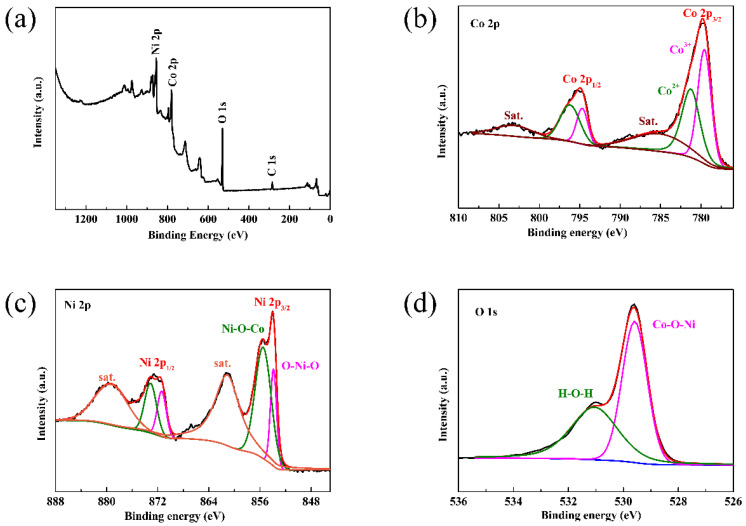
(**a**) Survey spectrum, (**b**) Co 2p, (**c**) Ni 2p and (**d**) O 1s for CoNiO_2_ of XPS spectra.

Further confirmations of surface elemental composition were done using X-ray photoelectron spectroscopy (Figure 5). Figure 5a shows the full scan spectrum of CoNiO_2_, in which Co, Ni and O elements coexist. According to the Co 2p spectra (Figure 5b), the binding energies at 795.0 eV and 779.8 eV correspond to the peaks of Co 2p_1/2_ and Co 2p_3/2_, respectively, while the satellite peaks of 803.3 eV and 785.9 eV are associated with its satellites [29]. A correlation was found between Ni 2p_3/2_ and Ni 2p_1/2_, as indicated by the characteristic peaks of Ni 2p in Figure 5c located at 854.0 eV and 872.8 eV, and the relevant satellite peaks were also observed at 861.1 eV and 879.5 eV [30]. There are also two characteristic peaks in the O 1s spectrum (Figure 5d); one at 529.6 eV represents oxygen species in the CoNiO_2_ phase and the other represents oxygen-containing residuals like H-O-H. Based on the above characterizations, the hierarchical CoNiO_2_ microspheres are synthesized successfully.

**Figure 6:**
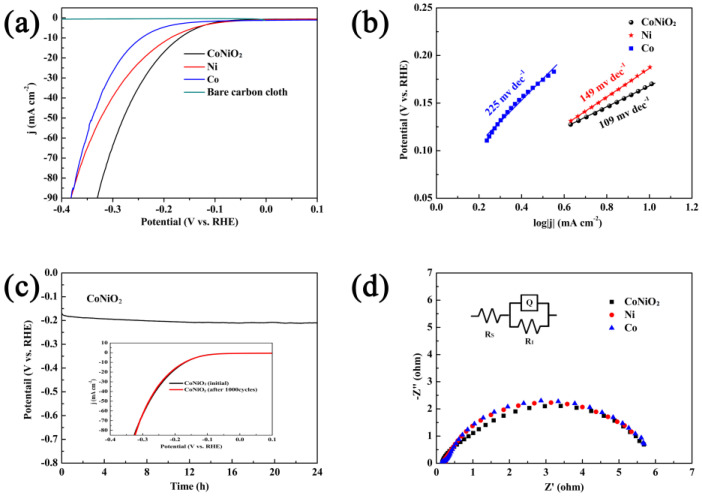
(**a**) Polarization curves and the corresponding (**b**) slopes of Tafel of CoNiO_2_, metallic Ni and metallic Co, respectively. (**c**) Chronopotentiometry curve of CoNiO_2_ at 10 milliampere square centimeter for 24 h. This inset shows the polarization curves of the material before and after a 1000 cycles period. (**d**) Nyquist plots of the CoNiO_2_, metallic Ni and metallic Co at −300 mV overpotential.

Using a typical three-electrode setup that contained a concentration of 1 M KOH, hierarchical CoNiO_2_, metallic Ni and metallic Co were investigated for their electrocatalytic activities. Figure 6a illustrates that HER polarization curves were measured at 1 mV s^−1^ with IR correction for CoNiO_2_, Ni, Co and bare carbon cloth. The CoNiO_2_ catalyst has a lower onset overpotential of 94 mV when compared to Ni and Co catalysts, and has a faster current response when the potential goes up, while pure carbon cloth has negligible electrocatalytic activity. Furthermore, CoNiO_2_ has only an overpotential of 170 mV at 10 mA cm^−2^, which is lower than that of Ni (188 mV) and Co (244 mV), showing that it has a superior catalytic activity compared to these two metals. The HER performance of CoNiO_2_ is also superior to other Co-Ni composite materials reported previously (Table 1), which benefits from the nanoflower structure providing more active sites and the synergistic effect.

We further explored the inherent properties of different catalysts by evaluating the reaction kinetics of HER using Tafel plots as a way. Figure 6b illustrates that the Tafel slope of CoNiO_2_ is 109 mV dec^−1^, a value lower than both Ni (149 mV dec^−1^) and Co catalysts (225 mV dec^−1^). As indicated by the Tafel slope of 109 mV dec^−1^, the hydrogen evolution process on CoNiO_2_ is governed by the Volmer-Heyrovsky mechanism, and the lower value is indicative of the more favorable characteristics of its kinetic behavior toward hydrogen evolution.

The stability and durability of the as-synthesized CoNiO_2_ are also evaluated in alkaline condition. Figure 6c shows that the chronopotentiometry curve shows no obvious degradation after 10 h at 10 mA cm^−2^. As can be seen from Figure 6c (inset), the polarization curve starts to overlap the initial curve almost completely, and this maintains the current density of 98.2% even after 1000 continuous sweeping cycles. All the above results prove that CoNiO_2_ has excellent HER activity, along with great stability.

Using electrochemical impedance spectroscopy (EIS), we conducted an analysis with an overpotential of −300 mV in order to better understand the HER kinetics of CoNiO_2_, metallic Ni and metallic Co. Figure 6d shows the corresponding Nyquist plots and Figure 6d inset shows the equivalent circuit model. It is clearly observed that CoNiO_2_ presents the lowest charge transport resistance (R_ct_), reflecting its better electronic conductivity. Specifically, CoNiO_2_, Ni and Co possess R_ct_ values of 5.13, 5.27 and 5.35 Ω, respectively. Smaller R_ct_ values indicate that CoNiO_2_ owns quicker charge transfer than other samples. During the HER process of CoNiO_2_, the electrons deliver directly and efficiently between the catalyst and the solution, contributing well to activity.

**Figure 7:**
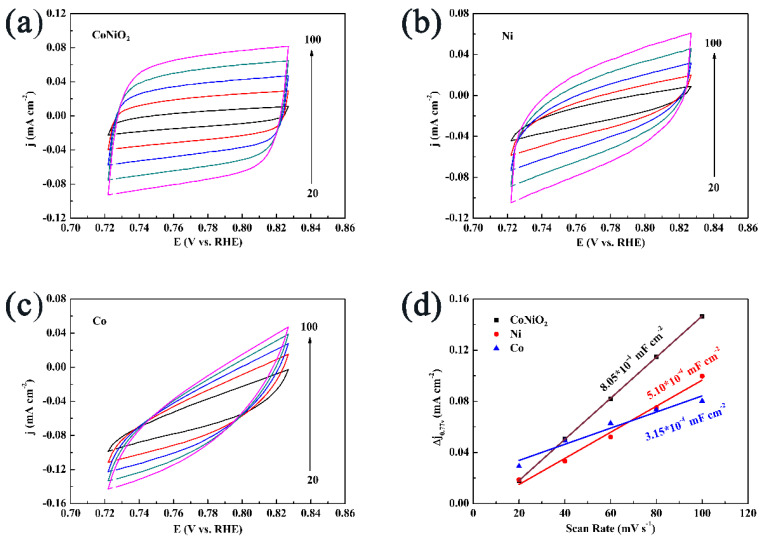
The CV curves of (**a**) CoNiO_2_, (**b**) metallic Ni and (**c**) metallic Co. (**d**) The double−layer capacitance values CoNiO_2_, metallic Ni and metallic Co in 1 M KOH.

An electrochemical surface area with a higher value typically means there are more active sites in the electrochemical layer, which results in higher HER activity. In addition, the electrochemical surface area varies with the capacitance of the double layer, so that it is proportional to the surface area. There are several CV curves that can be seen in Figure 7a–c related to electrocatalysts that have been tested with a scan rate that ranges from 20 to 100 mV s^−1^, namely CoNiO_2_, metallic Ni and metallic Co in an alkaline electrolyte. At 0.77 V, we calculated the current density difference between the anode and cathode (∆j = J_anode_ − J_cathode_). It can be obtained twice the value of double-layer capacitance (C_dl_) by fitting the scanning rate and ∆j together. As you can see in Figure 7d, CoNiO_2_ has a large C_dl_ value (8.05 * 10^−4^ mF cm^−2^) compared to metallic Ni (5.10 * 10^−4^ mF cm^−2^) and metallic Co (3.15 * 10^−4^ mF cm^−2^). There was a noticeable difference between CoNiO_2_ and all the other materials investigated in respect of their electrochemical surface area and HER activity in the alkaline electrolyte based on the results.

**Figure 8:**
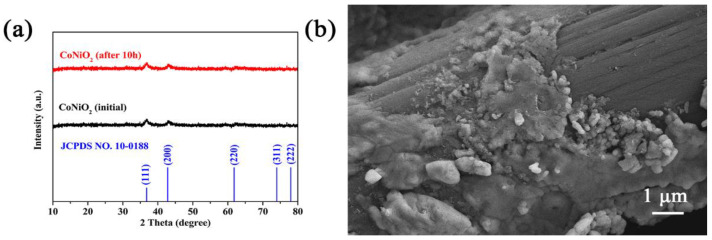
(**a**) XRD patterns of before and after a 10 h test of CoNiO_2_ in an alkaline electrolyte. (**b**) SEM images of after a 10 h test of CoNiO_2_ in an alkaline electrolyte.

The composition of the CoNiO_2_ process was examined by XRD following the HER for 10 h. As shown in Figure 8a, the XRD curves before and after 10 h test of CoNiO_2_ in an alkaline electrolyte have been displayed. The results show that all the peaks after the 10 h test have remained approximately the same as they were before the test, which proves that the CoNiO_2_ in an alkaline electrolyte is a very stable compound. SEM images (Figure 8b) were taken of the same samples after 10 h of HER in an alkaline electrolyte, which indicate that the morphologies after the test are nanoparticles agglomerated compared to the ones before the test.

**Figure 9:**
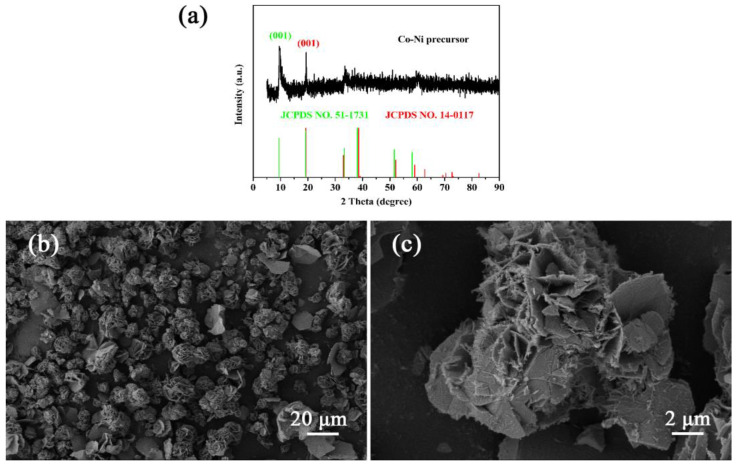
(**a**) XRD patterns of the cobalt-nickel precursor. (**b**,**c**) SEM images of the cobalt-nickel precursor.

As shown in Figure 9a, the characteristic peak of the cobalt-nickel precursor located at 9.5° corresponds to the (001) planes of Co(OH)_2_ (JCPDS No. 51-1731), and is located at 19.3° (001) planes of Ni(OH)_2_ (JCPDS No. 14-0117). As shown in the SEM image (Figure 9a,b), the cobalt-nickel precursor presents the flower-like microsphere structure.

**Figure 10:**
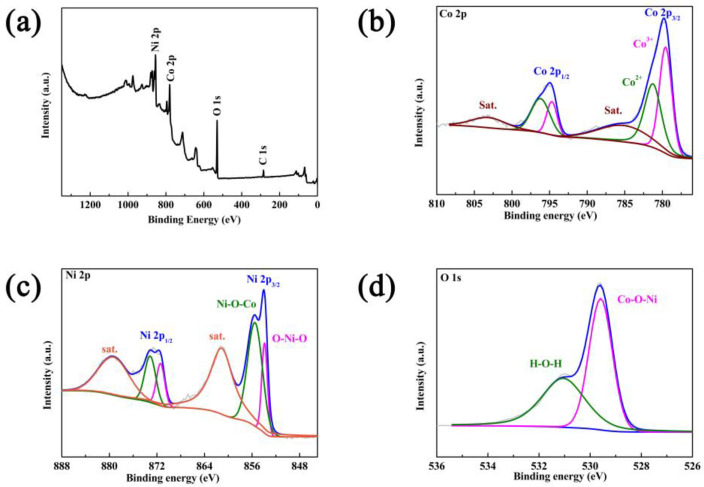
(**a**) Survey spectrum, (**b**) Co 2p, (**c**) Ni 2p and (**d**) O 1s for CoNiO_2_ post-HER of XPS spectra.

The surface elemental composition of the CoNiO_2_ process was examined by XPS following the post-HER. As shown in Figure 10, the XPS curves of CoNiO_2_ of the post-HER in an alkaline electrolyte have been displayed. The results show that the chemical surface composition of post-HER is the same as before and that there is no difference, which proves that the CoNiO_2_ in an alkaline electrolyte is a very stable compound.

## 4. Conclusions

In summary, it can be concluded that the hierarchical mesoporous CoNiO_2_ has been successfully prepared, and that this CoNiO_2_ can be used as a catalyst in water to catalyze the HER reaction. In HER, CoNiO_2_ only has an onset potential of 94 mV and an overpotential of 170 mV at 10 mA cm^−2^ due to the synergistic effect, compared with metallic Co and metallic Ni. Meanwhile, CoNiO_2_ presents the improved kinetic characteristics with a Tafel slope (109 mV dec^−1^). The enhanced reaction kinetics of CoNiO_2_ can be attributed to the high surface area, and are beneficial for contacting with electrolytes and enhancing the transport of charges. Additionally, CoNiO_2_ shows very good long-term stability and can withstand for over 10 h. The excellent electrocatalytic activity with high stability makes CoNiO_2_ promising for application in HER.

## Figures and Tables

**Table 1:** In 1 M KOH, CoNiO_2_ electrocatalyst performance in HER was compared with that of cobalt- and nickel-based electrocatalysts.

Catalyst	Current Density (mA·cm^−2^)	Potential (mV)	Reference
NiFe-LDH/NiCo_2_O_4_	10	192	[31]
NiCo_2_O_4_ nanowies	10	294	[31]
Ni/NiO	10	226	[32]
Air- NiCo_2_O_4_	10	226	[33]
Ni@NiO	10	192	[34]
Ni_3_S_2_@Ni(OH)_2_	10	237	[35]
CeO_2_/Co(OH)_2_	10	317	[36]
CoO-3D graphen	10	360	[37]
Co-Ni hybrid oxides	10	378	[38]
MoSx@NiO	10	406	[39]
CoNiO_2_	10	170	This work

## Data Availability

Due to privacy, data is not provided at this time.
